# Optimization of Pressurized Alkaline Hydrolysis for Chemical Recycling of Post-Consumer PET Waste

**DOI:** 10.3390/ma17112619

**Published:** 2024-05-29

**Authors:** Izotz Amundarain, Asier Asueta, Jon Leivar, Katrin Santin, Sixto Arnaiz

**Affiliations:** GAIKER Technology Centre, Basque Research and Technology Alliance (BRTA), Parque Tecnológico de Bizkaia, Edificio 202, 48170 Zamudio, Spain; asueta@gaiker.es (A.A.); leivar@gaiker.es (J.L.); katrinsantin@gmail.com (K.S.); arnaiz@gaiker.es (S.A.)

**Keywords:** poly(ethylene terephthalate) waste, chemical recycling, hydrolysis, monomer

## Abstract

Addressing the environmental impact of poly(ethylene terephthalate) (PET) disposal highlights the need for efficient recycling methods. Chemical recycling, specifically alkaline hydrolysis, presents a promising avenue for PET waste management by depolymerizing PET into its constituent monomers. This study focuses on optimizing the pressurized alkaline hydrolysis process for post-consumer PET residues obtained from packaging materials. Post-consumer PET packaging waste was chemically recycled by means of an alkaline hydrolysis reaction in a 2 L pressurized reactor under varying conditions of the NaOH/PET ratio and temperature. The reaction’s progress was monitored by sampling the liquid phase hourly over a four-hour period. The obtained products were purified, with a focus on isolating terephthalic acid (TPA). Higher temperatures (150 °C) resulted in superior TPA yields (>95%) compared to lower temperatures (120 °C). The NaOH/PET ratio showed minimal influence on the TPA yield. The optimal conditions (T = 150 °C; NaOH:PET = 2) were identified based on TPA yield and reaction cost considerations. This study demonstrates the feasibility of pressurized alkaline hydrolysis for PET recycling, with optimized conditions yielding high TPA purity and efficiency.

## 1. Introduction

Plastics have cemented their status as indispensable materials in contemporary society, with their utilization undergoing a remarkable surge over the past few decades. Recent data indicate that global plastic production has more than doubled since the year 2000, reaching approximately 368 million metric tons in 2019 [1]. This upward trajectory has been accompanied by a proportional increase in plastic waste generation, posing significant environmental challenges. Notably, plastic waste generation has outpaced population growth in recent years, highlighting the urgency of addressing this issue [2].

While plastics constitute a relatively small proportion of urban solid waste by weight (approximately 7%), their low density allows them to occupy a disproportionately large volume, contributing to visual pollution and environmental degradation [3]. Of particular concern is the persistence of plastics in the environment, with estimates suggesting that certain plastic items can take hundreds to thousands of years to decompose fully [4]. This persistence, coupled with an inadequate waste management infrastructure and practices, has led to the accumulation of plastic debris in terrestrial and marine ecosystems worldwide, with profound implications for biodiversity and human health [5].

Among the various types of plastics, poly(ethylene terephthalate) (PET) has emerged as a widely used thermoplastic polymer, valued for its versatility, durability, and transparency. PET finds extensive applications in packaging, including bottles for beverages, food containers, and textiles [6]. However, the widespread adoption of PET has also contributed to its prevalence in the waste stream, necessitating effective strategies for its management and recycling.

In response to the mounting challenges posed by plastic waste, there has been a growing emphasis on sustainable waste management practices, with recycling playing a pivotal role. Recycling offers multiple benefits, including resource conservation, energy savings, and waste diversion from landfills and incinerators. However, despite the potential advantages of recycling, current recycling rates for plastics remain relatively low, with significant room for improvement [7].

Mechanical recycling represents the most common method of recycling PET currently employed. This process involves shredding and reprocessing PET waste into pellets or flakes, which can then be used to manufacture new products. While mechanical recycling offers a viable solution for certain PET applications, its effectiveness is limited by factors such as contamination, degradation of material properties, and technological constraints [8].

In addition to mechanical recycling, there is growing interest in advanced recycling technologies, including chemical recycling, which enables the conversion of plastic waste back into its constituent monomers or other value-added products. Chemical recycling holds promise for achieving higher-quality recycled materials and closing the loop in the plastics value chain. However, challenges related to scalability, cost-effectiveness, and environmental impact must be addressed to realize the full potential of this approach [9].

In recent years, several methods of depolymerization of PET have been developed with the aim of obtaining terephthalic acid (TPA) [10], dimethyl terephthalate (DMT) [11] or bis(2-hydroxyethyl) terephthalate (BHET) [12], all of which are possible monomers to produce new polyesters. The exact monomer obtained from such depolymerization will depend on the chemical agent used for the breakdown of the chain, according to which the different reactions can be classified as follows: methanol, hydrolysis, glycolysis, ammonolysis, and aminolysis. In hydrolysis, the reaction of PET with water allows the breakdown of the chain into TPA and ethylene glycol (EG), being able to develop in an acidic, basic or neutral medium. Hydrolysis in an acidic medium is usually performed with sulfuric acid, and the large amount required makes the process substantially more expensive [13]. Furthermore, the use of this acid makes it necessary to supply the equipment with an extra resistance to corrosion [14]. On the other hand, the main disadvantage of neutral hydrolysis is the use of large excess reagents, as well as the need for high temperatures and pressures [15]. In addition, this process produces co-products such as oligomers, TPA derivatives or even cyclic trimers [16]. The alkaline hydrolysis reaction involves the treatment of polyester with an aqueous NaOH solution (between 4% and 20% by weight) [17]. The literature suggests that the most recent studies on the alkaline hydrolysis of PET studies were conducted at temperatures ranging from 80 °C to 220 °C [18]. The latest patent for alkaline hydrolysis reported the application of sodium hydroxide to produce TPA from PET, and the reaction temperature range was 130–150 °C [19]. Under appropriate reaction conditions, a disodium salt of TPA is formed and, by acidification, the TPA is recovered from the solution as a precipitate.

Although previous research has identified hydrolysis as one of the most suitable chemical recycling processes for PET recycling, applications developed from these findings mainly focus on relatively clean PET materials with a known composition and rely on the use of phase transfer catalysts (PTC) that can be difficult to recover and also expensive. Furthermore, the present work deals with real PET waste that is currently being generated and landfilled or incinerated in large quantities, so this paper aims to present a technically feasible solution based on the principles of the circular economy, thus allowing the manufacture of new value-added products and closing the cycle of PET materials through the application of a chemical recycling process. Nevertheless, the consulted literature suggests that no complex PET waste streams have been studied for the pressurized and alkaline hydrolysis of PET until now. Therefore, this work aims to explore the feasibility of the pressurized hydrolysis of complex PET waste, without the use of a catalyst, and study the progression of the reaction by means of the TPA yield.

In view of the need to improve and optimize chemical recycling processes to make them as effective and feasible as possible, the present study tries to explore the optimization of the process with a lower amount of NaOH and a lower temperature compared to previous results, in order to reduce operating costs and to make the process more profitable and sustainable in terms of material and energy consumption, always maintaining TPA yield values higher than 90%. Therefore, this study aims to investigate the hydrolysis of PET as a key step in the chemical depolymerization process for PET recycling. By optimizing the hydrolysis process, we seek to enhance the efficiency and sustainability of PET recycling, thereby contributing to the transition towards a circular economy.

## 2. Experimental

### 2.1. Materials

**Figure 1:**
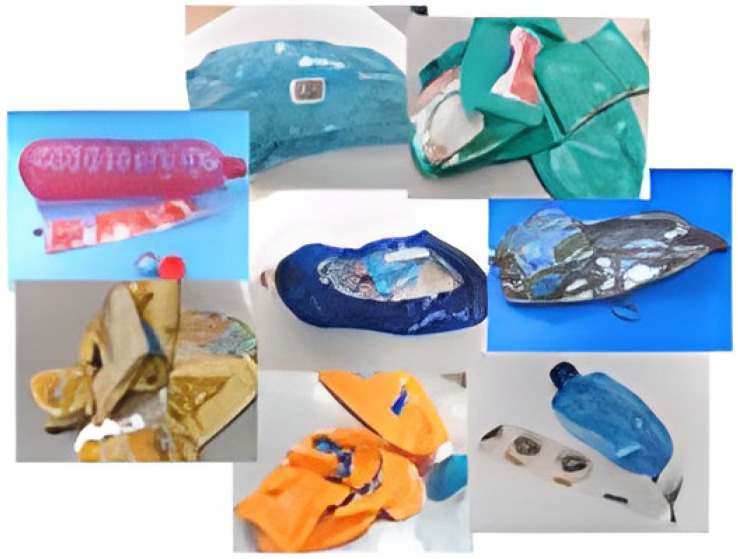
Post-consumer PET packaging.

**Figure 2:**
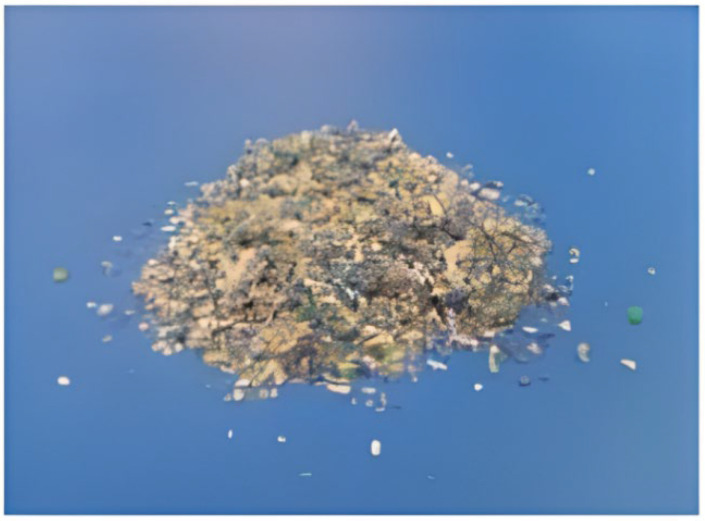
Waste under study conditioned.

Waste from post-consumer PET packaging was supplied by Ecoembes (Madrid, Spain) as shown in Figure 1. In Figure 2, the waste can be seen once conditioned with a particle size of 1 mm. To obtain a representative sample, the residue was subjected to a process based on the quart stack method, achieving the homogenization of the original sample and reducing the sample volume (ASTM C 702).

### 2.2. Pretreatment of Post-Consumer PET

The monomer purity and yield of the process can be improved if the waste sample is treated before the hydrolysis reaction. First, the sample was crushed and the different materials (carton, paper, etc.) were removed. Moreover, the metallic fraction was eliminated by a magnetic separator. Finally, the sample was milled to reach a particle size of 1 mm diameter.

### 2.3. Pressurized and Alkaline PET Hydrolysis

**Figure 3:**
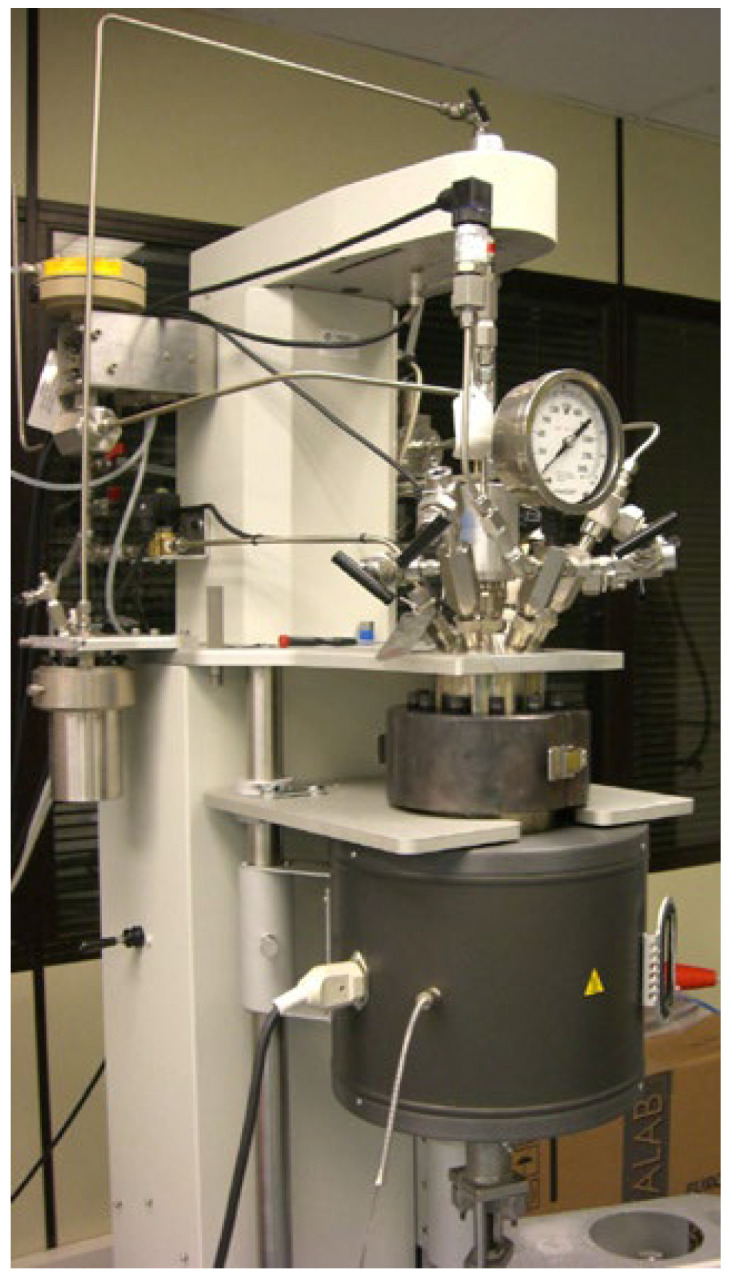
Parr reactor used throughout the experiment.

The reaction system which was used for the depolymerization of the PET waste was a 2 L agitated-tank Parr Instrument reactor (Figure 3). This reactor incorporates a control and monitoring system in order to optimize the operation conditions. The experimental procedure was the same in all reactions, varying only the dissolution volume of NaOH 0.5 M and operating temperature (Table 1). After feeding into the reactor a 40 g sample of waste PET and the required amount of the dissolution, the reaction was carried out for a total time of four hours, assuming as zero time the instant at which the temperature reached the value determined for each test. During the reaction, a 50 cm^3^ sample of the liquid phase was extracted every hour for the determination of TPA yield at every moment. The obtained products, PET monomers, were in a solution together with the PET and the unreacted NaOH, so further purification was necessary.

### 2.4. Purification of Obtained Products

For the purification of products, a pressure filtration unit was used, which has a nitrogen outlet on top. On the other hand, a stove and a furnace were also used to remove the moisture of both filters and obtained product. As for the reagents used, 37% HCl (Panreac, Barcelona, Spain) was used to obtain a solution of 12 M, and 0.1 N KOH-ethanolic was purchased from Sigma Aldrich-Merck (Darmstadt, Germany).

Once the reaction was complete, the reactor content was divided into two phases: a solid phase, due to the non-reacted sample fraction, and a liquid phase, in which the monomers generated in the hydrolysis and part of the NaOH solution that had not reacted were present. After a first filtration, the non-reacted solid phase required washing with distilled water and drying in the stove for four hours at a temperature of 80 °C. After this time, it was weighed at room temperature to calculate the total sample conversion.

PET depolymerization results in TPA and EG. However, the purification of the EG is a complicated task, so this study focused only on the purification of the TPA, once obtained in the form of disodium salt. In addition, the recovery rate of EG obtained after the hydrolysis of PET was much lower than that obtained for TPA [20], as a result of the catalytic effect of the carboxylic protons generated from the TPA itself on the dehydration reactions in the EG, which provides acetaldehydes and diethyl glycol.

Thus, all samples collected every hour were first filtered in order to separate any residual PET still unreacted. The filtrate liquid was treated with HCl 12 M to cause TPA precipitation from disodium salt, as shown in Equation (1). The solid TPA obtained was again filtered and washed with distilled water to remove any impurities present. Finally, it was introduced into the stove at 80 °C for 4 h and once cooled it was again weighed.

(1) $${\mathrm{Na}}_{2}\mathrm{TPA}+2\mathrm{HCl}\to \mathrm{TPA}+2\mathrm{NaCl}$$

### 2.5. TPA Characterization

After the purification of TPA, it was possible to determine its purity via titration with KOH-ethanolic acid and phenolphthalein acting as indicators. So, the proportion of TPA present in the collected solid was determined by comparing the results with a calibration line. Finally, the clarification resulting from this second filtration, in which the EG was located, required special management since it was a mixture of several components. Also, TPA yield could be calculated by Equation (2). When the reaction was completed, the percentage of PET that had reacted could be quantified based on the mass of fed and non-reacted PET.

(2) $$\eta_{\mathrm{TPA}}\left(\%\right)=\frac{\mathrm{mol}{\mathrm{TPA}}_{\mathrm{formed}}}{\mathrm{mol}{\mathrm{TPA}}_{\left(\eta \mathrm{TPA}=1\right)}}\times \;100=\frac{\mathrm{mol}{\mathrm{TPA}}_{\mathrm{sample}}\times \frac{{\mathrm{volume}}_{\mathrm{reaction}}}{{\mathrm{volume}}_{\mathrm{sample}}}}{\mathrm{mol}{\mathrm{PET}}_{0}}\times \;100$$

Due to the pigments and colorants added during PET processing, PET depolymerization can also lead to a colored TPA monomer, the polymerization of which in turn produces a colored rPET [21,22]. Furthermore, the presence of color in rPET can also limit its application and viability in comparison to commercial PET. Hence, a characterization of the color of TPA produced after PET hydrolysis was also carried out using a CM-2300d model spectrophotometer with a wavelength range between 360 nm and 740 nm. The CIE 1976 L*a*b* (CIELAB) color space was used, which defines the color of an object through three parameters: L* (100 = white; 0 = black), a* (positive = red; negative = green; 0 = gray) and b* (positive = yellow; negative = blue; 0 = gray). TPA was also identified via Fourier transform infrared spectroscopy (FTIR). FTIR measurements were recorded using Shimadzu software in an IRAffinity equipment, MIRacle10 (diamond prism/ZnSe), in the wavenumber range of 600–4000 cm^−1^, with a resolution of 4 cm^−1^ and with 20 scans.

## 3. Results

### 3.1. Obtained Results

**Figure 4:**
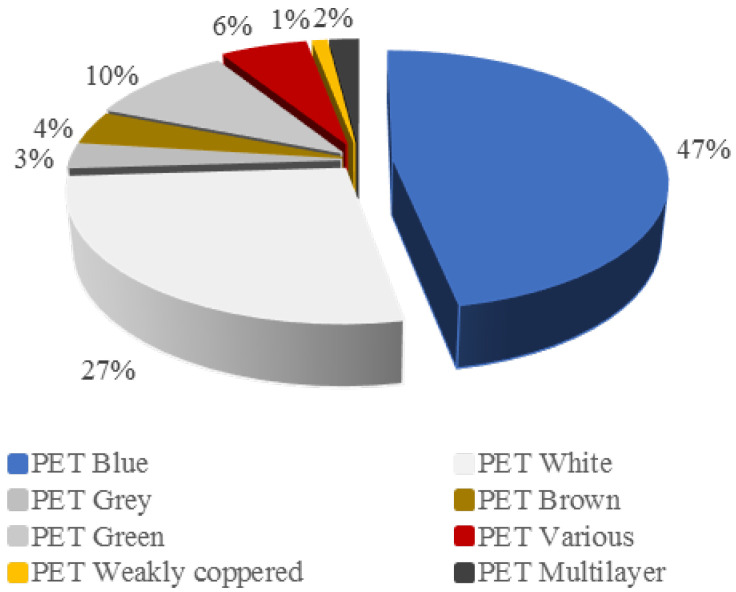
Characterization regarding the color of the sample.

**Figure 5:**
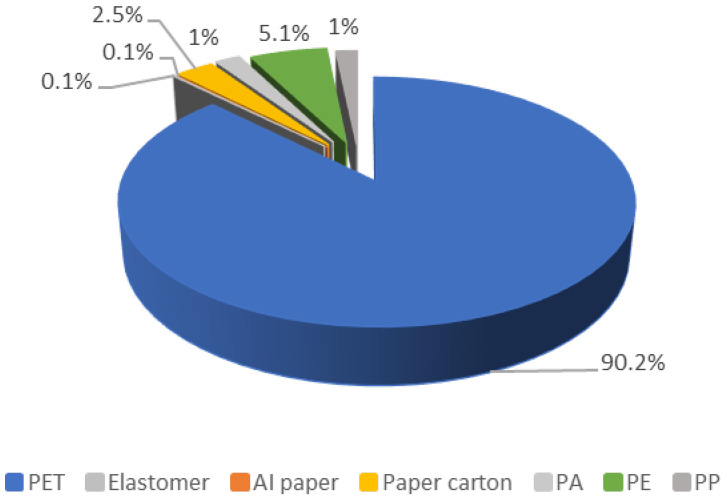
Characterization of the types of polymers present in the sample.

The characterization results for the post-consumer PET waste samples are represented in Figure 4 and Figure 5. The color of the sample is important because of the possible coloration that can be present in the TPA monomer. Nearly half of the studied samples were blue PET. Also, together with the PET, other plastics such as PE, PP and PA were present in the analyzed samples as impurities for the hydrolysis reaction.

TPA yield was taken as a limiting parameter when rejecting the different combinations of the operating conditions by setting the value of 90% as the minimum acceptable. This value is calculated using Equation (2). Table 2 shows the results obtained for each of the samples collected in the first six reactions.

### 3.2. Effect of the Operating Variables on the TPA Yield

**Figure 6:**
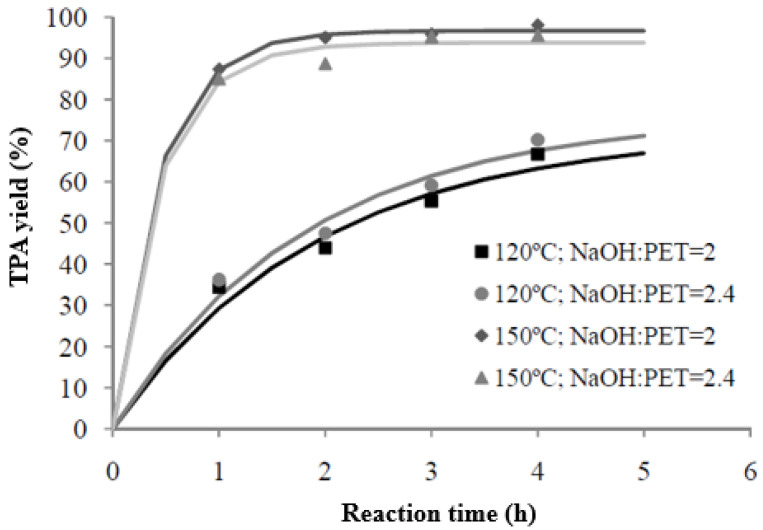
TPA yield vs. reaction time. Influence of NaOH:PET.

Once the reactions were carried out and the yields were determined for all the samples, the results were represented in order to observe the effect of the variation in some of the parameters on the output variable, TPA yield. This determined the operating conditions to be studied in subsequent tests. The yield values obtained in each reaction are represented in Figure 6. After this, exponential adjustment was performed through the computer application CurveExpert 1.3, which helped us to see yield trends over time.

#### 3.2.1. Temperature Effect

It can be seen in Figure 6 that temperature is a major factor in the reaction, with the yield of the monomer of interest increasing with temperature. At 120 °C, the maximum yield exceeds 70%, while in the tests performed at 150 °C, yields above 95% are obtained. Thus, it is observed that an increase of 30 °C produces an increase in yield close to 25%.

#### 3.2.2. Effect of NaOH:PET Molar Relation

Unlike the changes observed with the temperature variation, the NaOH/PET ratio hardly changes the yield obtained. At 120 °C, an increase in the solvent ratio, from the stoichiometric ratio (=2) to a higher ratio (=2.4), only leads to an increase of about 4% in the yield obtained. In the case of 150 °C, higher yields are obtained for the stoichiometric ratio. In any case, the difference found in the influence of the correlation did not exceed 3%, meaning that it can be said that the yields obtained are barely affected by the NaOH/PET ratio. In addition, the higher the temperature, the more important its effect and, consequently, the lower the concentration of the solvent present in the reactor.

#### 3.2.3. Estimation of Experimental Error

**Figure 7:**
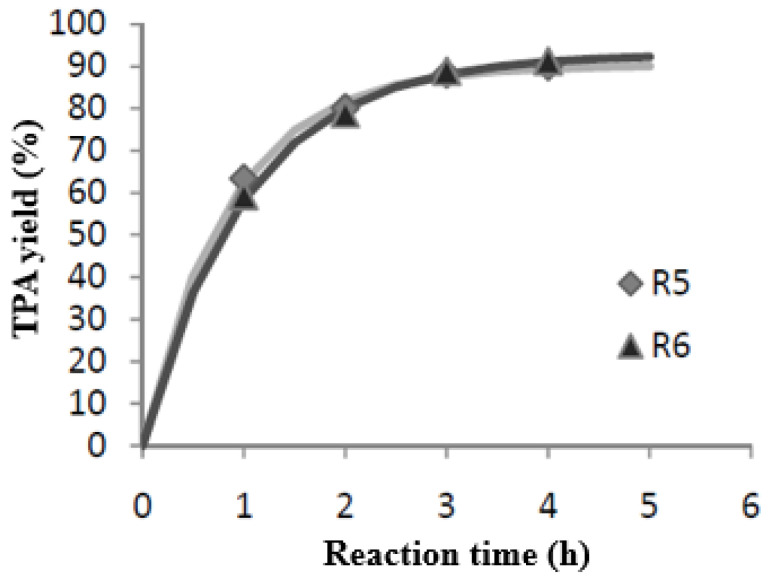
Representation of the two central points.

Once the influence of each variable on the yield had been determined, a reaction with intermediate operating conditions in each variable was performed twice. Thus, the experimental error could be estimated, defining this as the result of the ratio between the standard deviation and the arithmetic mean of the results obtained for each sample in both reactions. Figure 7 shows the least variation obtained for both duplicates. Using the mean for the four points studied, an experimental error of 1.9% was obtained, with a standard deviation value of 1.33. On the other hand, as indicated above, the average TPA yield obtained under these conditions was 90.58%, slightly higher than the limit value previously established.

### 3.3. Estimation of Optimal Reaction Conditions

Once the effect of the variables studied had been established and the new starting point (T = 135 °C and NaOH:PET = 2.2) had been determined, the improvement of these conditions was established as a new objective. To this end, the aim was to reduce reaction costs as much as possible, taking care not to reduce the yield. We studied the possibility of maintaining the temperature at 135 °C while reducing the NaOH/PET ratio to the stoichiometric level. However, this combination did not achieve returns above 83.94%, so this point was dismissed for non-compliance with the specifications.

**Figure 8:**
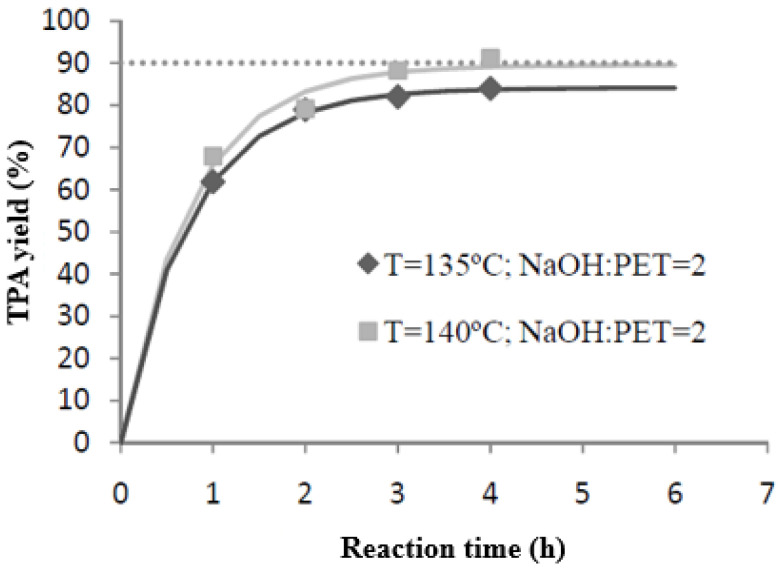
Estimation of optimal reaction conditions.

For the last reaction, therefore, the temperature was found to be a variable of great influence, as shown above. Thus, this time the solvent/sample ratio was maintained at the stoichiometric level and the temperature was increased to 140 °C. As can be seen in Figure 8, this time, the results were satisfactory as they met the stipulated requirements (η_TPA_ = 91.18%).

**Figure 9:**
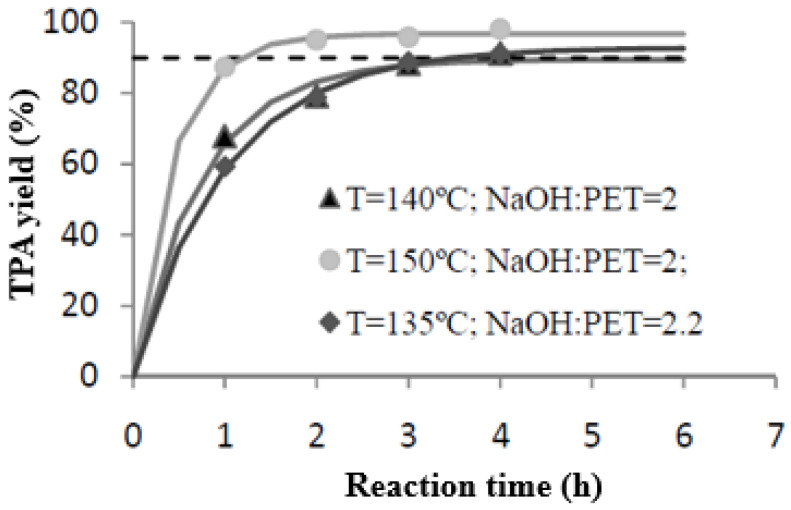
Selection of possible optimal operating conditions. Reactions and conditions considered in the economic study of the reaction stage.

There are two reactions that meet the marked specifications: R8 (T = 140 °C; NaOH:PET = 2) and R5/R6 (T = 135 °C; NaOH:PET = 2.2). In order to establish preferences for a particular reaction and thinking about the execution of this process at an industrial scale, we assessed which of the situations would generate higher costs in the operation. To make this estimate, only the costs that differed between the reactions to be studied were calculated—that is, the cost generated by the use of the NaOH reagent and the cost due to the energy invested in heating the reactor to the operating temperature. When making this calculation, a new factor to consider arose: the reaction time. This is a fundamental parameter, since its reduction decreases the energy required in the process and, in consequence, the cost. Therefore, all reaction conditions and times that managed to produce a yield exceeding 90% were considered. However, some of them were dismissed due to the presumably higher cost due to the greater use of reagents. Therefore, the study was reduced to three parameters reflected in Figure 9 and Table 3.

According to recent reports from Ecoembes, in 2022, about 1,627,313 tons of packaging were recovered in Spain [23]. That addresses a collection and sorting for recycling rate of 70.1% according to Eurostat [24]. The estimate of the cost was performed on the remaining 29.9% of packaging waste. In the calculations, it was assumed that this fraction had a composition similar to that obtained for the sample. On the one hand, the cost generated by the consumption of NaOH is based on the price provided chemical providers, subject to possible fluctuations according to different options.

On the other hand, the cost of the energy invested in heating the reactor must also be calculated. For this purpose, we started from the usual dimensions of an industrial discontinuous depolymerization reactor agitated tank. According to the literature, this reactor usually has a volume of 2.5 m^3^, and maintains a height/diameter ratio of 3:1. Since about 25% of reactors are usually oversized to ensure results, height values of 1 m and 3 m were taken for this study. So, knowing the coefficient of heat transmission of stainless steel (17 W/m^2^ °C) and the reactor transmission area (A = π × D × h), we calculated the power required to reach the desired temperature, according to Equation (3).

(3) $$P=17\;\left(\frac{W}{m^{2}\;{}^{\circ}C}\right)\times \pi \times 1\left(m\right)\times 3\times T({}^{\circ}C)$$

Moreover, considering a reactor downtime of about 2 h per 4 h of the reaction, invested in cooling, unloading, cleaning and reloading the reactor, 16 h of reaction per day was assumed, meaning a total of 5600 h per year (350 working days) for reactions lasting four hours and half that (2800 h) for those lasting two. Also, energy cost was considered. Table 4 shows the costs calculated for the three reactions considered.

It can be seen how the least viable option is that given by the central point (R5/R6), since the high cost of the reagent, NaOH, makes the total cost of the reaction considerably increase. The other two reactions, R3 and R8, have the same cost as regards the use of reagent. However, the reaction time required for the first is half that required for the last, so despite having to heat the reactor to 150 °C, the energy cost is reduced by about half and, with it, the total reaction cost also reduces. In addition, it is advisable to reduce the depolymerization time to below 3 h, because higher contact times increase the presence of coloring substances in the resulting TPA and may reduce its purity [25]. Therefore, the optimal operating conditions for the reaction under study are finally established, as shown in Table 5.

Regarding the purity of the TPA obtained, only one of the recovered samples was analyzed, assuming that since the procedure was identical for all reactions, the purity would also be the same. Thus, the product was evaluated with KOH-ethanolic 1 N in triplicate (P1, P2 and P3) and compared with a previously calculated calibration line, obtaining purity percentages of 99.13%, 97.85% and 98.67%, respectively. Therefore, an average product purity of 98.5% was assumed, meeting the reaction expectations.

### 3.4. Comparison of Sample Performance with Virgin PET

**Figure 10:**
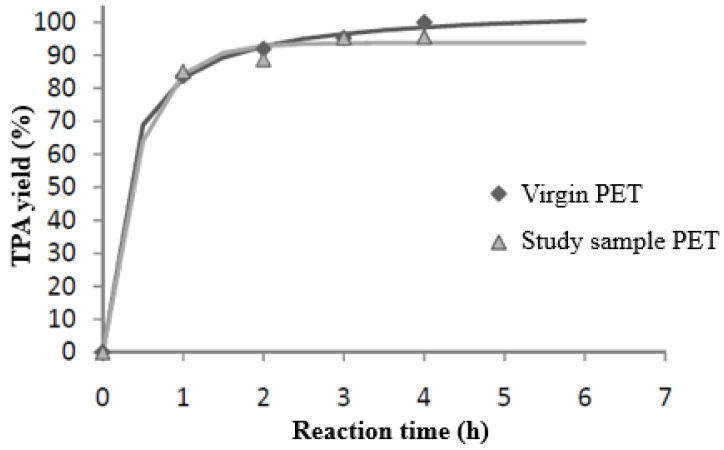
TPA yield vs. reaction time. Comparison of study sample performance with virgin PET sample (T = 150 °C; NaOH:PET = 2.4).

As can be seen in Figure 10, under equal operating conditions, slightly higher yields were obtained from the virgin PET sample. In the case of virgin PET, four hours of the reaction was enough to obtain 100% yields. However, in the case of the sample with different compounds, maximum yields of 95.5% were obtained at the same reaction time. This may be due to the influence of these materials on the reaction, whether in the presence of secondary reactions or different reaction kinetics [26].

Previous studies showed the first-order kinetics of the hydrolysis reaction in an alkaline medium of the PET without a catalyst [15]. On this occasion, the residue studied was not a sample of virgin PET. Therefore, the kinetics of the reaction could be altered by the presence of improper materials. Performing a short kinetic study of the results obtained under the conditions determined as optimal for the sample of virgin PET, T = 150 °C and NaOH:PET = 2.4 ratio, a value of the apparent kinetic constant of 0.8843 m^3^/kmol h was obtained. It should be noted that this is not a real value, as product yield values were used instead of the conversion rate. However, it provides an idea of the possible influence of the presence of impurities in the sample on kinetics. If this value is compared with that obtained with virgin PET (2.076 m^3^/kmol h), kinetics can be expected to be faster when the sample is composed entirely of PET.

### 3.5. Characterization of Obtained TPA

**Figure 11:**
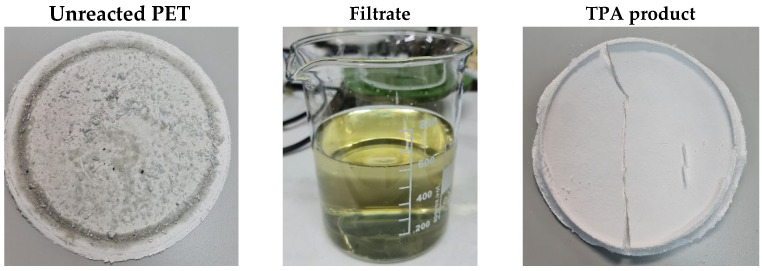
Graphic illustrations of the unreacted PET cake, filtrate, and TPA product.

Figure 11 illustrates the unreacted PET tray cake, the separated unreacted filtrate, and the neutralized solid TPA product after filtration. Despite the intense color of the filtered product, after neutralization with acid and filtering, the final product loses much of its color.

**Figure 12:**
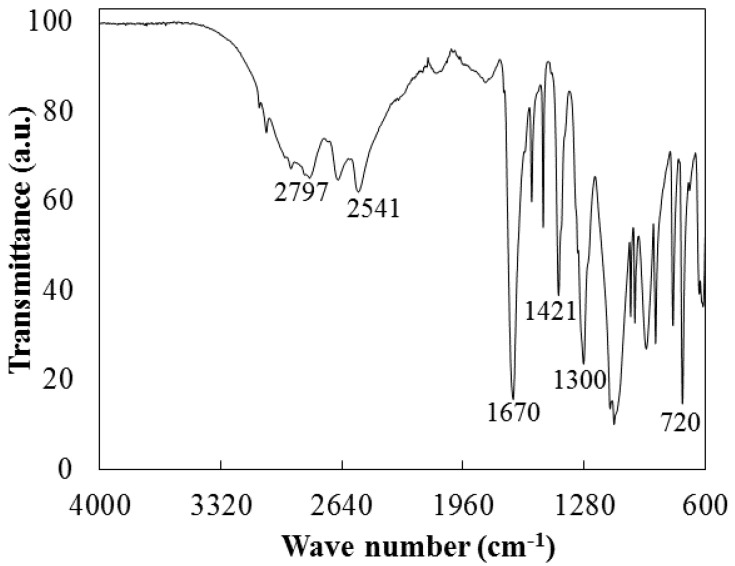
FTIR spectra of the TPA product.

The transformation of Na_2_TPA into TPA after acidification was confirmed by FTIR analysis (Figure 12). Characteristic absorption peaks were observed at 3018 cm^−1^, 2804 cm^−1^ and 2549 cm^−1^, corresponding to carboxylic groups. The signals at 1693 cm^−1^, 1300 cm^−1^ and 1420 cm^−1^ are related to the carbonyl group. The characteristic peaks corresponding to the aromatic rings were noted in the region of 700–800 cm^−1^ [27].

Furthermore, the difference in color between the TPA produced from the depolymerization of the PET tray and the reference TPA turned out to be adequate, with low difference values under the investigated reaction conditions, which is indeed a clear advantage of the subsequent polymerization of TPA to colorless plastics, especially required in the food sector. The CIELAB parameters are shown in Table 6.

## 4. Conclusions

Plastics, particularly PET, have become indispensable in modern society, leading to a significant increase in plastic waste production. PET’s desirable properties make it widely used in packaging for various industries, but its environmental impact is a concern due to the high volume of production and the reliance on non-renewable resources like oil. To address these concerns, efforts have been made to recycle PET, with mechanical recycling being the most common method. However, mechanical recycling has limitations in producing high-quality recycled PET.

Chemical recycling, particularly depolymerization, shows promise in overcoming the limitations of mechanical recycling by breaking down PET into its original monomers for reuse. Hydrolysis, especially alkaline hydrolysis, is a prominent method for PET depolymerization, but challenges such as high cost, co-product formation, and energy consumption exist. This research focused on optimizing the pressurized alkaline hydrolysis process for post-consumer PET packaging waste, considering variables like temperature and the NaOH/PET ratio.

The results showed that higher temperatures significantly increased TPA yield, while the NaOH/PET ratio had a minimal effect. The study identified the optimal conditions (T = 150 °C, NaOH/PET = 2) for PET depolymerization, considering both yield and cost factors. The purity of the obtained TPA met expectations, with an average purity of 98.5%. Comparison with virgin PET showed slightly lower yields from the sample, possibly due to the presence of impurities affecting the reaction kinetics. Despite challenges, the optimized pressurized alkaline hydrolysis process offers a viable solution for recycling post-consumer PET packaging waste, contributing to environmental sustainability.

## Figures and Tables

**Table 1:** Operating conditions for each reaction.

Experiment Number	NaOH:PET (mol)	Temperature (°C)
R1	2	120
R2	2.4	120
R3	2	150
R4	2.4	150
R5	2.2	135
R6	2.2	135
R7	2	135
R8	2	140

**Table 2:** Yield to the monomer of interest obtained for each of the analyzed samples.

Experiment Number	T (°C)	NaOH/PET	TPA Yield (%)			
			1 h	2 h	3 h	4 h
R1	120	2.0	34.38	43.98	55.30	66.68
R2	120	2.4	36.23	47.50	59.17	70.22
R3	150	2.0	87.33	95.05	95.82	98.08
R4	150	2.4	85.05	88.74	95.23	95.59
R5	135	2.2	63.40	80.14	88.17	89.90
R6	135	2.2	59.29	78.78	88.89	91.27

**Table 3:** Selection of possible optimal operating conditions.

Time (h)	NaOH:PET	Temperature (°C)
2	2	150
4	2.2	135
4	2	140

**Table 4:** Cost generated by the use of NaOH and the energy expenditure invested in heating the reactor for each of the reactions considered.

t (h)	NaOH:PET	T (°C)	Cost per NaOH (EUR)	Energy Cost (EUR)	Total (EUR)
2	2	150	3,345,232,000	6,729,291	3,351,961,291
4	2.2	135	3,679,755,200	12,112,725	3,691,867,925
4	2	140	3,345,232,000	12,561,344	3,357,793,244

**Table 5:** Estimated optimal values for different operating variables.

T (°C)	NaOH:PET	Stirring Rate (rpm)	Reaction Time (h)
150	2	250	2

**Table 6:** CIELAB color parameters and color difference with respect to the standard TPA for the produced TPA product.

	Reference TPA	Produced TPA
L*	98.75	95.14
a*	−0.10	−0.18
b*	1.10	2.27

## Data Availability

The data presented in this study are available on request from the corresponding author.
